# Response of triploid Atlantic salmon (*Salmo salar*) to commercial vaccines

**DOI:** 10.1016/j.fsi.2019.12.070

**Published:** 2020-02

**Authors:** Lynn Chalmers, Herve Migaud, Alexandra Adams, Luisa M. Vera, Elsbeth McStay, Ben North, Chris Mitchell, John F. Taylor

**Affiliations:** a Institute of Aquaculture, University of Stirling, Stirling, FK9 4LA, UK; b PHARMAQ (part of Zoetis), Unit 15 Sandleheath Industrial Estate, Fordingbridge, Hampshire, SP6 1PA, UK

**Keywords:** Triploid, Vaccination, Adhesions, Vertebral deformity, Performance, Side-effects

## Abstract

While triploid Atlantic salmon represent a practical and affordable solution to the issues associated with sexual maturation in the salmonid aquaculture industry, empirical evidence suggests triploids are more susceptible to disease and vaccine side-effects than diploids. With vaccination now part of routine husbandry, it is essential their response be studied to confirm their suitability for commercial production. This study tested the response of triploid and diploid Atlantic salmon to vaccination with commercially available vaccines. Triploid and diploid Atlantic salmon siblings were injected with one of three commercial vaccines (or sham-vaccinated) and monitored for performance throughout a commercial production cycle. Sampling at smolt and harvest was undertaken along with individual weight and length assessments through the cycle. Antibody response to *Aeromonas salmonicida* vaccination was similar in both ploidy, with a positive response in vaccine-injected fish. For both adhesions and melanin, analysis found that higher scores were more likely to occur as the anticipated severity of the vaccine increased. In addition, for adhesion scores at smolt and melanin scores at smolt and harvest, triploids were statistically more likely to exhibit high scores than diploids. Triploids maintained a significantly higher body weight during freshwater and until 11 months post-seawater transfer, with diploids weighing significantly more at harvest. Growth, represented by thermal growth coefficient (TGC), decreased in both ploidy as the severity of adhesions increased, and regression patterns did not differ significantly between ploidy. Vertebral deformity prevalence was consistently higher in triploids (smolt 12.3 ± 4.5%; harvest 34.9 ± 5.9%) than diploids (smolt 0.8 ± 0.5%; harvest 15.9 ± 1.9%), with no significant difference between vaccine groups in each ploidy. This study demonstrates that triploids respond as well to vaccination as diploids and provides further supporting evidence of triploid robustness for commercial aquaculture.

## Highlights

• Antibody response to *Aeromonas salmonicida* vaccination was similar in both ploidy.
• Higher adhesion and melanin scores more likely to occur in triploids and as the anticipated severity of vaccine increased.
• Triploids heavier in freshwater while diploids were significantly heavier at harvest.
• Thermal growth coefficient significantly related to increasing severity of adhesions.
• Higher deformity prevalence in triploids, with no significant effect of vaccine group.

## 1 Introduction

Disease is considered one of the most significant constraints to the continued development and success of Atlantic salmon aquaculture, with substantial economic losses caused by increased mortalities, downgrading at harvest and treatment use [1,2]. The aquaculture industry has employed numerous strategies to prevent disease outbreaks in intensive farming including improved biosecurity procedures (using reliable egg/fish stocks; water quality monitoring; disinfection of vehicles and equipment) and the implementation of diagnostic tools for early pathogen detection [[2], [3], [4]]. However, many pathogens form part of the ubiquitous and otherwise benign aquatic microfauna and only become pathogenic when the fish are subjected to unfavourable conditions acting as stressors [2,[5], [6], [7]]. The treatment for many of these pathogens has been limited to antibiotics and chemotherapeutics but, over the years, extensive use has resulted in the development of pathogen resistance to the once effective products [[8], [9], [10], [11], [12]]. With prevention regarded better than a cure, vaccination has become the single most important tool for disease control in the aquaculture industry, with research since the late 1930's resulting in the production of numerous effective aquatic vaccines [[13], [14], [15], [16], [17]].

However, vaccination may reduce growth and result in side-effects, including adhesions and increased vertebral deformity prevalence which impact negatively on fish health and welfare as well as production quality [[18], [19], [20], [21], [22]]. Adhesions form adjacent to the injection site and, if severe, can connect organs firmly to the peritoneum and cause damage to the muscle upon removal [[22], [23], [24]]. Vertebral deformities can be evident prior to vaccination but inflammation around the spine, as a result of vaccination and the associated handling has been suggested to aggravate vertebral deformities post-vaccination [21,[25], [26], [27]].

Triploid Atlantic salmon (*Salmo salar*) have long been considered as a method to prevent early sexual maturation and the associated negative impacts on fish performance prior to harvest in aquaculture [28,29]. Over the last decade, numerous advances have been made in triploid research, with studies demonstrating triploid salmon have many different requirements to diploids, including a lower thermal optimum and higher requirements for key nutrients including phosphorus and histidine [[30], [31], [32], [33], [34], [35], [36]]. However, there continues to be limited data regarding the response of triploid Atlantic salmon to disease and disease treatments with historical and empirical reports suggesting an increased susceptibility in triploids. Following experimental challenge with *Renibacterium salmoninarum*, diploid and triploid Atlantic salmon were found to be similarly susceptible to the pathogen [37], with challenge by *Aeromonas salmonicida* eliciting similar levels of mortality and antibody responses between ploidy [38]. In response to challenges with viral (salmon alphavirus) and parasitic (*Lepeophtheirus salmonis*; *Neoparamoeba perurans*) pathogens, infection rates and severity were similar in both ploidy, with comparable mortality observed during parasite challenges [[39], [40], [41]]. While these studies demonstrate similar susceptibility to controlled disease challenges, few studies to date have investigated the response of triploid Atlantic salmon to vaccination.

Vaccination with ALPHA-JECT 2-2 (PHARMAQ AS) produced similar adhesion scores in diploid and triploid Atlantic salmon and had no significant effect on weight [38]. Intraperitoneal administration of MINOVA 6 Vet (Norvax®, Intervet International B.V., Boxmeer, Netherlands) negatively impacted on growth in S1+ diploid and triploid Atlantic salmon smolts, although no other side-effects were assessed in this study [42]. A follow up study by Fraser et al. [43] using diploid and triploid Atlantic salmon with both S0+ and S1+ smolt groups, again, showed a negative effect of vaccination with MINOVA 6 Vet on weight. Significantly higher adhesion scores were also found in triploid S0+ smolts compared to their diploid sibling group, while no ploidy effect was found in S1+ smolts. Within diploids and triploids under both smolt regimes, vaccination did not have a direct impact on prevalence or severity of vertebral deformities [43]. Between ploidy, however, triploid S1+ smolts had significantly higher levels of vertebral deformity than their diploid siblings, while no ploidy effect was observed in the S0+ smolts. The authors hypothesised that, while S0+ production is considered as a risk factor due to the known correlation between increased growth rates at high temperatures and vertebral deformity development [44,45], the elevated temperatures in S0+ production may have impeded growth rates in triploids thus alleviating the risk of fish developing severe vertebral deformities.

These studies highlight that vaccination can impact the performance and health of triploids, as well as diploids, although they were relatively limited in their assessment of adhesions and deformities as side-effects with a focus on only two vaccines. Considering the benefits associated with the use of triploids in commercial aquaculture production and the almost universal adoption of vaccination of farmed Atlantic salmon, it is essential that studies be undertaken to elucidate the tolerance of triploids to the vaccination process as well as the associated side-effects.

The aim of this study was to compare the response of triploid and diploid Atlantic salmon to a range of commercially available vaccines, including an “accidental double-dose”, and assess side-effects in terms of performance, the development of abdominal adhesions and occurrence of vertebral deformities.

## 2 Materials and methods

### 2.1 Fish stock and history

**Fig. 1:**
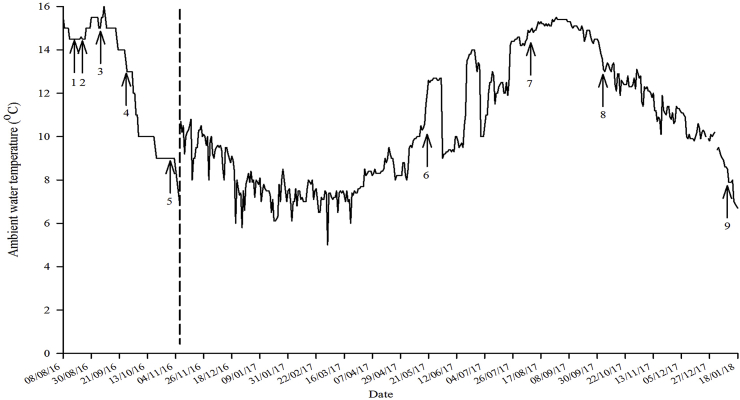
Temperature profile for the duration of the experimental trial (August 2016 to January 2018). Dashed line indicates seawater transfer. Black arrows indicate 1) P.I.T tagging, 2) “winter” (LD12:12) photoperiod applied, 3) vaccination, 4) LL photoperiod re-applied to induce smoltification 5) smolt sampling, 6) May weight assessment, 7) August weight assessment, 8) October weight assessment, 9) harvest sampling.

On 23^rd^ October 2015, eggs and milt were stripped from unrelated 2-sea winter Atlantic salmon broodstock (20 dams & 5 sires; strain PD-Strong, Mowi, Tveitvag, MOWI Norway). Following fertilisation, half of each egg batch was subjected to a pressure shock of 655 bar for 6.25 min, applied at 37.5 min post-fertilisation at 8 °C (TRC-APV; Aqua Pressure Vessel, TRC Hydraulics Inc., Dieppe, NB, Canada) to induce triploidy. Water hardened eggs were then incubated in six upwelling silos (3 ploidy^−1^) at 6.7 °C (temperature range: 5.4 °C–7.0 °C) until automatic sorting. Survival at sorting was 88% and 85% for diploid and triploid ova, respectively. Eyed ova were delivered to Howietoun Hatchery, Sauchieburn, Stirling on 16^th^ December 2015 (353 °D post-fertilisation). Eggs were then incubated in mesh trays in a flow-through system at 7.0 ± 0.3 °C in 6 × 300 L square fibreglass tanks (5000 eggs ploidy^−1^, 1 m^2^, 0.3 m depth) in darkness until first feeding (26^th^ February 2016). Mortality from egg receipt to first feeding was 5.1% and 6.5% for diploids and triploids, respectively. At first feeding, fry were reared under constant light and fed a commercial diet (diploids - Inicio Plus; triploids – Inicio-TriX, BioMar UK), distributed by automatic feeders (Arvo-Tec Oy, Finland). When approximately 0.5 g, fry were divided evenly between 12 × 300 L to reduce stocking density. On 11^th^ May 2016, all fry (~1.22 g) were transferred from the hatchery to the Niall Bromage Freshwater Research Facility (NBFRF), Buckieburn, and stocked into two flow-through 28 m^3^ holding tanks (1 ploidy^−1^), and exposed to a continuous light (LL) photoperiod prior to the start of the experiment. Tanks were maintained at ambient temperature (10–15 °C) (Fig. 1). Specific feeding rates (% tank biomass per day) were adjusted automatically according to predicted growth and daily temperature, and pellet size (0.5–2.0 mm) increased with fish size. Prior to the start of the experiment (8^th^ August 2016), both stock tanks were graded (5^th^ August 2016) using a box grader (12 mm bar, STERNER AquaTech) to remove the bottom grade.

### 2.2 Experimental design

On 8^th^ August 2016, diploid (18.6 ± 0.3 g) and triploid (25.4 ± 0.4 g) Atlantic salmon were stocked into six lightproof 1.6 m^3^ circular (2 m diameter) tanks (3 tanks ploidy^−1^, 350 fish tank^−1^) and maintained under an LL photoperiod. On 17^th^ August 2016, all fish (350 fish tank^−1^) were individually tagged using Trovan® (Trovan Ltd, UK) and Pet ID (Pet ID Microchips Ltd., West Sussex, UK) passive integrated transponder (P.I.T) tags for identification throughout the trial. Mortality between P.I.T tagging and vaccination was 1.67% for diploids and 1.09% for triploids. Following tagging, all tanks were subjected to a “winter” (LD 12:12) photoperiod for 5 weeks, before being returned to LL (26^th^ September 2016) for ~400 °D to induce S0+ smoltification, prior to seawater transfer (7th – 8^th^ November 2016).

From 6th to 8^th^ September 2016 (average temperature 15.3 °C), all experimental fish were vaccinated (diploids: 44.2 ± 0.7 g; triploids: 57.8 ± 1.6 g; *p* = 0.002). All fish were anaesthetised with Tricaine (50 ppm, PHARMAQ, Norway) and then seventy fish per tank were intraperitoneally injected with either 0.02 M phosphate buffered saline (PBS; Group A), ALPHA JECT 2-2 (Group B), ALPHA JECT micro 6 (Group C), ALPHA JECT 6-2 (Group D) or ALPHA JECT 6-2 (double dose) (Group Dx2) using either a FISHJECTOR 0.1 ml or a MICROFISH 0.05 ml vaccine gun (Kaycee Veterinary Products Ltd., West Sussex, UK) depending on the dose (Table 1). At the time of vaccination, the body weight (g) and fork length (mm) of each fish was recorded. Following vaccination, all fish were returned to the appropriate trial tank for recovery and were maintained in these tanks for 52 days (622 °D) until sampling.

**Table 1:** Vaccine component details, delivered dose and anticipated adhesion score severity for the five experimental groups.

Group	Vaccine	Dose volume (μl)	Vaccine components	Anticipated adhesion score^a^
A	PBS	100	Acting as sham-vaccinated control group	0

B	ALPHA JECT 2-2	100	*Aeromonas salmonicida* subsp. *salmonicida*; IPNV serotype Sp	1.3

C	ALPHA JECT micro 6	50	*A. salmonicida* subsp. *salmonicida*; *Listonella anguillarum* serotypes O1 & O2a; *Vibrio salmonicida*; *Moritella viscosa*; IPNV serotype Sp	1.5

D	ALPHA JECT 6-2	100	*A. salmonicida* subsp. *salmonicida*; *L. anguillarum* serotypes O1 & O2a; *V. salmonicida*; *M. viscosa*; IPNV serotype Sp	2.2

Dx2	ALPHA JECT 6-2 (double-dose)	200	As above; acting as “accidental double dose injection” scenario	5

a Anticipated adhesion scores were calculated by PHARMAQ (Zoetis LLC, New Jersey, USA) using vaccine validation and test data, number of antigens and dose volume to give an increasing scale of severity, according to anticipated adhesion scores.

On 7th and 8th November, following “smolt” sampling, all remaining experimental Atlantic salmon (diploids 638; triploids 643) were transferred to triplicate sea-cages (5 m height x 5 m width x 5 m depth; net mesh 18 mm) at the Ardnish Feed Trial Unit (Lochailort, MOWI, Scotland, UK). Fish remained in the sea-cages under ambient light and water temperature (Fig. 1) until January 2018 at which time the trial was terminated and “harvest” sampling took place. Specific feeding rates (% cage biomass per day) were adjusted automatically according to predicted growth and daily temperature, and pellet size (3.5–12 mm in SW) increased with fish size and were distributed by automatic feeders (Arvo-Tec Oy, Finland). Fish were fed commercial seawater diets (diploids – BioMar CPK; triploids – BioMar CPK-TriX, BioMar UK) and daily waste feed was collected by siphon uplifts per pen.

### 2.3 Sampling

#### 2.3.1 Freshwater – Smolt

“Smolt” sampling was undertaken on 6 consecutive days from 31^st^ October 2016, with one full tank sampled per day, alternating between triploids and diploids. Fish were removed from the experimental tank and maintained in 300 L holding tanks for the duration of the sampling, with water aerated throughout (average temperature 8.6 °C). All fish were anaesthetised with 50 ppm Tricaine (PHARMAQ, PHARMAQ Ltd.) then the individual P.I.T tag number, body weight (g) and fork length (mm) recorded. Condition factor (K) [46] was calculated using: K = 100 x W/L^3^; where W is body weight (g) and L is fork length (mm). Thermal growth coefficient (TGC) [47] from vaccination to smolt, and then from smolt to harvest was calculated using: TGC = 1000 x (W_2_^1/3^ – W_1_^1/3^)/(T x t); where W_1_ is the initial weight for the period and W_2_ is the final weight, T is temperature in °C and t is time in days. As P.I.T tags were scanned, 25 fish vaccine group^−1^ tank^−1^ were culled (1000 ppm Tricaine and percussive blow to the head) for further sampling. Following sampling, all non-tissue sampled fish (approx. 225 tank^−1^) were returned to the experimental tank for recovery.

From culled fish, blood samples were obtained from the caudal vein using a non-heparinised needle and syringe and kept at 4 °C for 24 h. Samples were then centrifuged at 3000 g for 10 min before serum was collected and stored at −20 °C until determination of antibody response. The severity of intra-abdominal adhesions in the peritoneal cavity was scored visually according to the Speilberg scale [48]. For this, the peritoneal cavity was divided into three regions and a score given to each:

• Region 1: Anterior and anterior-dorsal parts of abdominal cavity including oesophagus, liver and anterior parts of the swim bladder
• Region 2: Posterior and posterior-dorsal parts of abdominal cavity including hind gut
• Region 3: Ventral region of abdomen close to the recommended injection site

For analysis, the highest score across the three zones was taken as the overall adhesion score. In addition, melanin deposits on the viscera were scored according to Pharmaq (Zoetis) (Table 2) [49].

**Table 2:** Scoring scheme for post-vaccination visceral melanin deposits [49].

Score	Visual appearance of abdominal cavity
0	No melanin in the abdominal cavity
1	Some faint melanin or small spots affecting small of the viscera
2	Moderate amounts on or within one or more organs
3	Extensive melanin deposits on viscera

Radiographs of each fish were then obtained using a device cabinet x-ray radiography unit (FaxinTron UltraFocus, Daax Ltd, USA; 24 kV, mAs: 5.0) and digital images were generated as dicom files for further analysis.

#### 2.3.2 Seawater sampling

Following transfer to seawater, individual weight (±1 g) and length (±1 mm) assessments were undertaken at the Ardnish Feed Trial Unit on 19^th^ May 2017 (192 days, 1586 °D post-transfer), 10^th^ August 2017 (283 days, 2722 °D post-transfer) and 6^th^ October 2017 (331 days, 3439 °D post-transfer). Fish were anaesthetised (50 ppm Tricaine) before individual P.I.T tag, weight and length were recorded. Fish were returned immediately to the sea-cage for recovery.

Final “harvest” sampling was undertaken on 6 consecutive days from 11^th^ January 2018. All remaining fish were terminally culled (1000 ppm MS-222 then percussive blow to the head). Following P.I.T tag identification, individual body weight and fork length was recorded. Condition factor (K) and TGC between smolt and harvest were calculated as previously described. Adhesions and melanin deposits were scored in all fish (200 sea-cage^−1^), as previously described. Furthermore, radiographs of 20 fish vaccine group^−1^ sea-cage^−1^ were obtained using a portable x-ray unit (Celtic SMR PX40 HF; 32 mAs; 40 kV) and AGFA XenOR 35CL detector plate, with digital images generated as Dicom files for pathology interpretation.

### 2.4 X-ray radiograph for vertebral deformities

Dicom images of each fish were analysed for vertebral deformities (RadiAnt Dicom Viewer software, Medixant, Poland). For each fish, total vertebrae number along with the location and deformed vertebrae (dV) pathology type [50] were recorded. Subsequently, pathologies were grouped together into decreased intervertebral space (type 1), compression (types 2, 3, 4 and 5), fusion (types 6, 7 and 8), radiodense (types 12 and 13), symmetry (types 17 and 19) and other (types 9, 10 and 11). For analysis, the vertebral column was divided into four regions (R): R1 (cranial trunk, V1 - 8), R2 (caudal trunk, V9 - 30), R3 (tail, V31 - 49) and R4 (tail fin, V50 – end) [51]. In consideration of the findings from Hansen et al. [44], x-rays were also classified according to severity in terms of number of dV. If 1–5 dV were observed, this was defined as “mild”, with ≥6 dV considered “moderate – severe and likely to affect welfare”. For statistical analysis, only fish classed as “moderate - severe” were assessed.

### 2.5 Enzyme-linked immunosorbent assay (ELISA)

The specific antibody response (IgM) of diploid and triploid Atlantic salmon to *Aeromonas salmonicida* was measured in serum samples using a modified version of the indirect ELISA method described by Adams et al. [52]. Ninety-six well microplates (Immulon 4HBX, Fisher Scientific, UK) were coated with 0.001% (w/v) poly-l-lysine (P8920, Sigma-Aldrich, USA) in coating buffer (50 μl well^−1^) for 60 min before being washed twice with low salt wash buffer (LSWB: 0.02 M Tris, 0.38 M NaCl, 0.05% Tween 20). Whole cell *A. salmonicida* ‘Hooke’ strain (1 × 10^8^ bacteria ml^−1^) was then added (100 μl well^−1^) and the plates incubated overnight at 4 °C. Following this, 0.05% (v/v) glutaraldehyde (G6403, Sigma-Aldrich, USA) in PBS was added to the antigen (50 μl well^−1^) and the plates incubated for a further 20 min at room temperature (RT). Plates were then washed a further 3 times with LSWB and post-coated with 3% (w/v) casein (250 μl well^−1^) for 2 h at RT to block non-specific binding sites. The post-coat was discarded before the addition of serum samples. Serum samples were diluted 1:50 with PBS, added to the microplates along with PBS in the negative and positive control wells (100 μl well^−1^) and incubated overnight at 4 °C. The microplates were washed with high salt wash buffer (HSWB: 0.02 M Tris, 0.5 M NaCl, 0.1% Tween 20), with a 5 min incubation on the last wash. Microplates were then incubated for 1 h at RT with mouse anti-Atlantic salmon IgM monoclonal antibody (F11, Aquatic Diagnostics Ltd., University of Stirling, Stirling, UK), diluted 1:33 with PBS (100 μl well^−1^). This is with the exception of the positive control wells which were incubated with rabbit anti-*A. salmonicida* polyclonal antibody (Aquatic Vaccine Unit, University of Stirling, Stirling, UK), diluted 1:1000 in PBS. The HSWB washes were then repeated before conjugates (anti-mouse IgG-HRP, A4416, Sigma-Aldrich, USA; anti-rabbit IgG-HRP, A6154, Sigma-Aldrich, USA), diluted 1:4000 with conjugate buffer, were added for 1 h (100 μl well^−1^). The HSWB washes were repeated and the reaction was developed with chromogen in substrate buffer (100 μl well^−1^). The reaction was stopped with 2 M H_2_SO_4_ (50 μl well^−1^) after 10 min then the absorbance was measured at 450 nm and values expressed as optical density (OD). The OD values for negative control wells were then multiplied by 3 and samples were considered positive if higher than this value.

### 2.6 Statistical analysis

Minitab software version 16 (Minitab Inc., Pennsylvania) was used to perform basic descriptive statistics and comparisons using a significance level of 5% (p = 0.05). Prior to analysis, datasets were checked for normality using the Anderson-Darling test. Mortality and vertebral deformity data as percentages were arcsine transformed for normality. Non-parametric tests were utilised if normal distribution was not achieved. For mortality, non-parametric Kruskal-Wallis and Dunn's multiple comparison post-hoc test were utilised (InStat. GraphPad Software, San Diego). For adhesion and melanin scores, ordinal logistic regression (OLR) was performed, with Vaccine Group A selected as the reference group for treatment analysis and diploids as the reference group for ploidy analysis. Antibody response, vertebral deformities and TGC were analysed using a GLM manipulated into a two-way ANOVA, with ploidy and vaccine group considered fixed factors and tank considered as a random factor. Post-hoc analyses were carried out using Tukey's multiple comparison tests with values considered significantly different at p-values < 0.05. Weight, length and condition factor were analysed using Analysis of Covariance (ANCOVA), with ploidy and vaccine group considered fixed factors, tank a random factor and weight at vaccination set as a covariate. Again, Tukey's multiple comparison test was used for post-hoc analysis. Statistical differences in the localisation of deformed vertebrae (%) between ploidy for each vertebral region were analysed by a one-way ANOVA. Regression analysis to detect linearity between adhesion score severity and TGC in both fresh- and seawater was carried out. Parallelism statistics were also performed using Excel (ANCOVA, Microsoft Office 2013, Washington, USA) to determine ploidy differences between the gradient of regression slopes.

## 3 Results

### 3.1 Mortality

Throughout the freshwater phase of the experiment (Sept to Nov 2016), mortality was less than 3% in all the groups, with no significant differences observed (Table 3A). In seawater (Nov 2016 to Jan 2018), mortality remained below 10% in the vaccine groups for both ploidy, with the exception of the triploid Dx2 group which experienced 16.8% mortality (Table 3B). Again, there was no significant effect of ploidy or vaccine on the mortality observed.

**Table 3:** Mortality (%) (mean ± SEM, n = 3) in the five vaccine groups for both diploid and triploid Atlantic salmon during the A) freshwater and B) seawater phases of the study.

GROUP	A	B	C	D	Dx2
A) Freshwater mortality (%)					
Diploid	1.4 ± 0.8	2.4 ± 1.3	2.4 ± 0.5	1.0 ± 1.0	2.0 ± 2.0
Triploid	2.4 ± 1.3	0.5 ± 0.5	1.0 ± 1.0	1.9 ± 0.5	1.6 ± 0.9
B) Seawater mortality (%)					
Diploid	8.4 ± 4.8	3.7 ± 1.9	5.4 ± 2.3	3.1 ± 1.9	4.4 ± 1.9
Triploid	3.1 ± 1.9	4.4 ± 2.5	7.6 ± 2.2	6.1 ± 1.9	16.8 ± 8.6

### 3.2 Antibody response

**Fig. 2:**
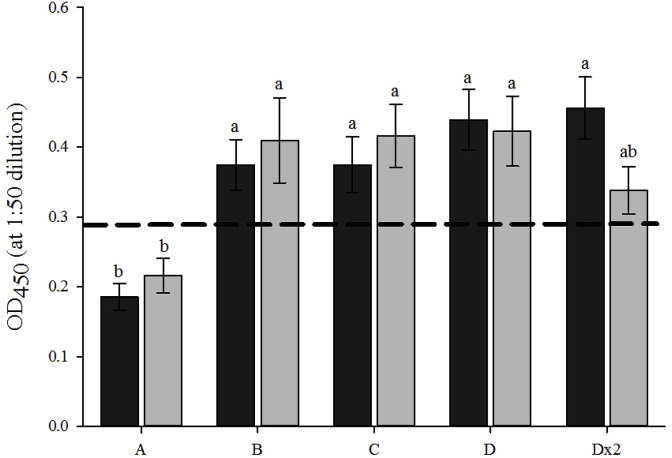
Antibody (IgM) response (OD450 nm, mean ± SEM, n = 3) against *Aeromonas salmonicida* at smolt in diploid (black) and triploid (grey) Atlantic salmon subjected to different commercial vaccines. Dashed line represents the value over which samples are considered positive. Significant differences between ploidy and vaccine group are indicated by different letters.

The antibody responses (specific IgM to *A. salmonicida*) of fish injected with a vaccine (Groups B, C, D, Dx2) were all positive (Fig. 2), while the antibody response of Group A (PBS) was negative. No significant effect of ploidy on antibody response was observed. Vaccine group had a significant effect on antibody response, with diploid and triploid Group A showing significantly lower antibody response than the other 4 groups, with the exception of the triploid Dx2 group.

### 3.3 Adhesions and melanin

**Fig. 3:**
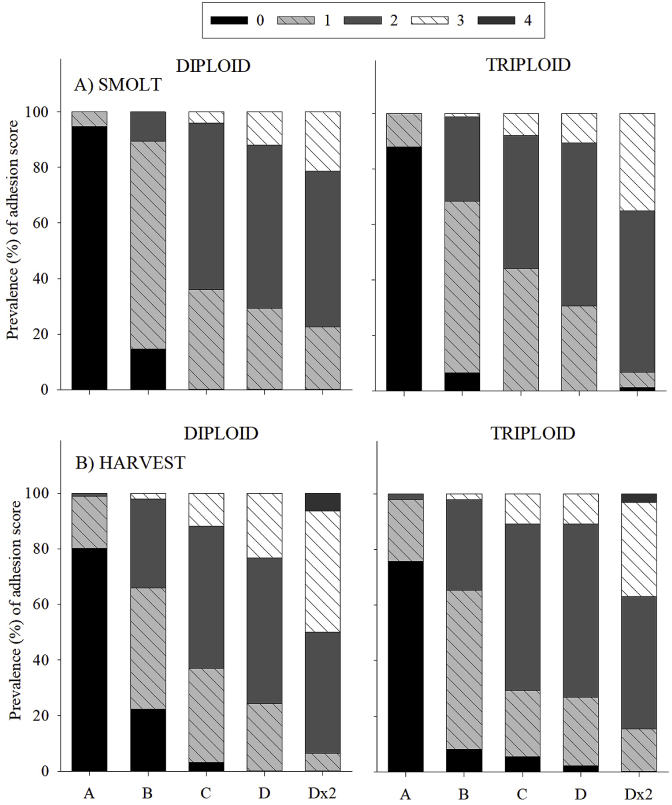
Prevalence (%) of each adhesion score (0, 1, 2, 3, 4) at A) smolt and B) harvest in diploid and triploid Atlantic salmon subjected to different commercial vaccines.

In comparison to the “reference group” (Group A) at both smolt and harvest, the likelihood that fish would experience “3” and “4” adhesion scores increased as vaccine group went from Group B to Group Dx2 (smolt: OLR coefficient = −4.8, −6.6, −7.1, −8.1, p = 0.000; harvest: OLR coefficient = −3.1, −4.5, −4.9, −6.3, p = 0.000) (Table 1; Fig. 3A and B). In this regard, lower scores (0–1) were more prevalent in Groups A and B while higher scores (2–4) were more prevalent in Groups C, D and Dx2.

In terms of ploidy differences at smolt, using diploids as the “reference group” suggested that higher adhesion scores were more likely to occur in triploids (OLR coefficient = 0.5; p = 0.004). This is evident in Fig. 3A as triploids present increased prevalence of “3” scores over the diploids, particularly in Group Dx2 (triploid 34.9%; diploid 21.3%). Conversely at harvest, triploids were not more likely to have higher scores than diploids (OLR coefficient = 0.05; p = 0.697). This is evident in Fig. 3B with triploids presenting lower prevalence of “3” (range: 2.1–33.5%) and “4” (3.2%) scores than diploids (“3” range: 2.1–43.6%; “4”: 6.4%).

**Fig. 4:**
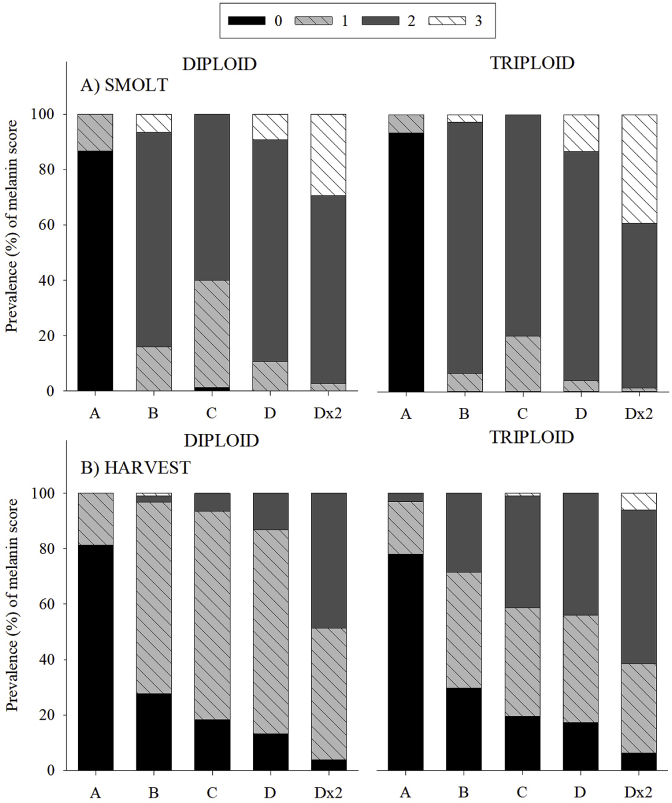
Prevalence (%) of each melanin score (0, 1,2, 3) at A) smolt and B) harvest in diploid and triploid Atlantic salmon subjected to different commercial vaccines.

In terms of melanin scores at smolt and harvest, in comparison to the “reference group” (Group A), as group increased from Group B to Dx2 so did the likelihood that fish would exhibit “3” scores (smolt: OLR coefficient = −9.0, −7.8, −9.8, −11.3; p = 0.000; harvest OLR coefficient = −2.4, −2.9, −3.2, −4.4; p = 0.000) (Table 1; Fig. 4). Thus, lower scores (0–1) were more prevalent in Groups A and B with higher scores (2–3) more prevalent in Groups C - Dx2.

Using diploids as the “reference group”, at both smolt and harvest findings suggested that higher adhesion scores were more likely to occur in triploids than diploids (smolt: OLR coefficient = 0.5, p = 0.009; harvest: coefficient = −0.7, p = 0.000). This is evident in Fig. 4A and 4B as triploids present increased prevalence of “3” scores (smolt range: 2.7–39.2%; harvest range: 1.1–6.2%) compared to diploids (smolt range: 6.7–29.3%; harvest range: 0–1.1%).

### 3.4 Growth

#### 3.4.1 Freshwater phase

**Fig. 5:**
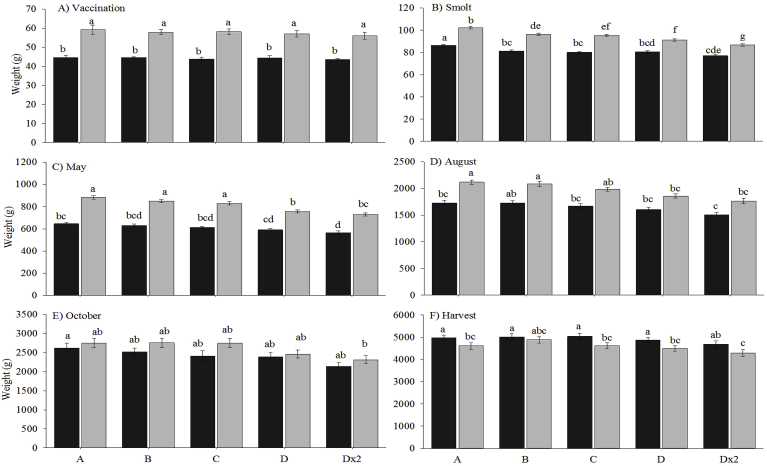
Weight (g) (mean ± SEM, n = 3) of diploid (black) and triploid (grey) Atlantic salmon in the five vaccine groups at A) vaccination, B) smolt (end of freshwater), C) May 2017, D) August 2017, E) October 2017 and F) harvest (Jan 2018) Significant differences between ploidy and vaccine groups are indicated by different letters.

At the point of vaccination, triploids (57.7 g ± 1.8 g) were significantly heavier than their diploid counterparts (44.2 ± 0.9 g) in all five vaccine groups (Fig. 5A). Within both ploidy, no significant differences were observed between vaccine groups.

At smolt, triploids (94.4 ± 1.1 g) were significantly heavier than their diploid counterparts (81.1 ± 0.9 g) in each vaccine group (Fig. 5B). Within diploids, Group A (86.4 ± 1.0 g) was significantly heavier than the other groups (79.8 ± 0.9 g), with no significant difference observed between groups B – Dx2. Similarly, triploid Group A (102.4 ± 1.3 g) was significantly heavier than the other 4 triploid groups, with Group B (96.4 ± 1.1 g) significantly heavier than Groups D (91.3 ± 1.1 g) and Dx2 (86.7 ± 1.0 g), and Group C (95.4 ± 1.2 g) heavier than Group Dx2.

**Fig. 6:**
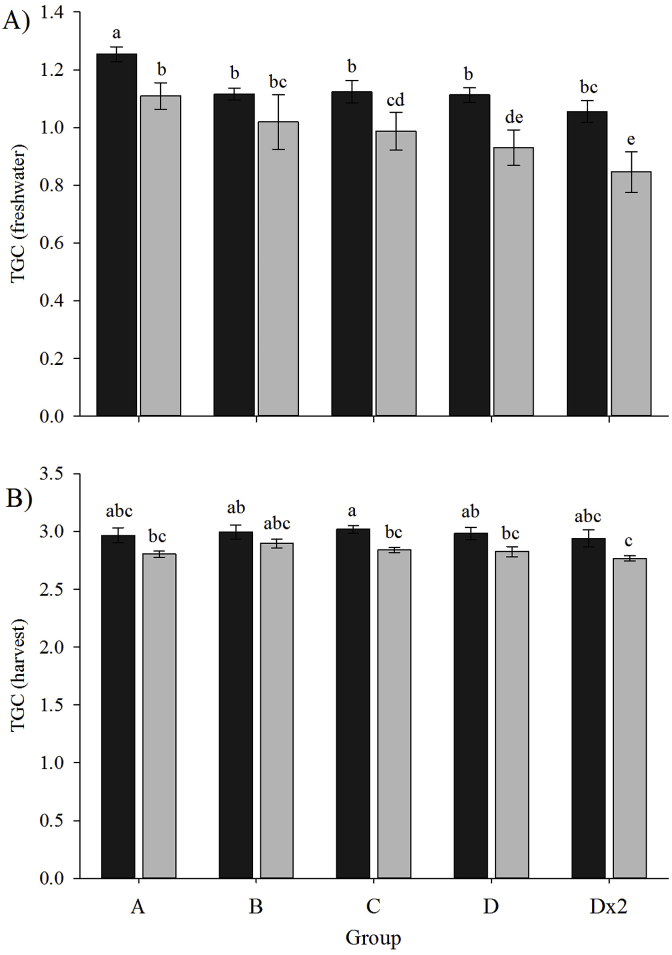
Thermal growth coefficient of diploid (black) and triploid (grey) Atlantic salmon in the five vaccine groups during A) freshwater (vaccination to smolt) and B) seawater (smolt to harvest) (mean ± SEM, n = 3). Significant differences between ploidy and vaccine group are indicated by different letters.

As reflected by smolt weight, ploidy and vaccine group significantly affected freshwater TGC (vaccination to smolt) (Fig. 6A). Diploid TGC (1.06–1.25) was significantly higher than triploids (0.85–1.11) in all groups, with the exception of Group B (diploid 1.12 ± 0.02; triploid 1.02 ± 0.09) (Fig. 6A). Within the diploids, TGC was significantly higher in Group A (1.25 ± 0.03) than the other four groups (1.06–1.12). In the triploids, TGC in Group A (1.11 ± 0.05) was significantly higher than in Groups C (099 ± 0.06), D (0.93 ± 0.06) and Dx2 (0.85 ± 0.07). The TGC of Group B (1.02 ± 0.09) was significantly higher than in Groups D and Dx2, with Group C TGC significantly higher than Group Dx2.

**Fig. 7:**
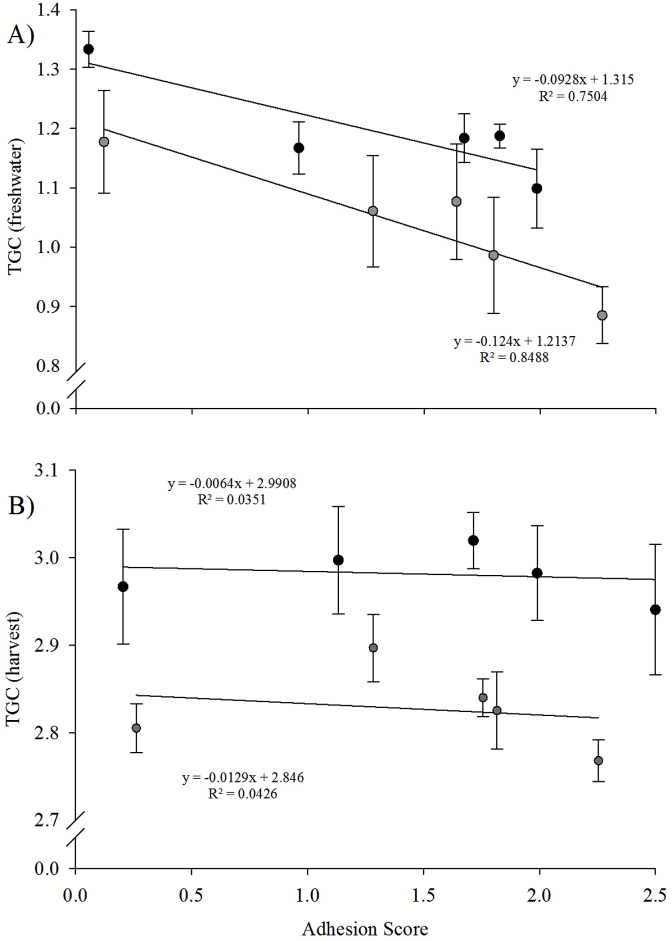
Linear regression of relationship between adhesion score and TGC in diploid (black) and triploid (grey) Atlantic salmon for the A) freshwater and B) seawater phases of the experiment.

A significant negative correlation (*p* = 0.02) was found between TGC and increasing adhesion score (Fig. 7A). Regression analysis showed that the vertical distance between the diploid and triploid slopes was not statistically significant (*p* = 0.18), with parallelism statistics indicating that slope gradient was statistically similar between ploidy (*p* = > 0.05) and thus that diploids and triploids showed similar responses to increasing “vaccine severity”.

**Fig. 8:**
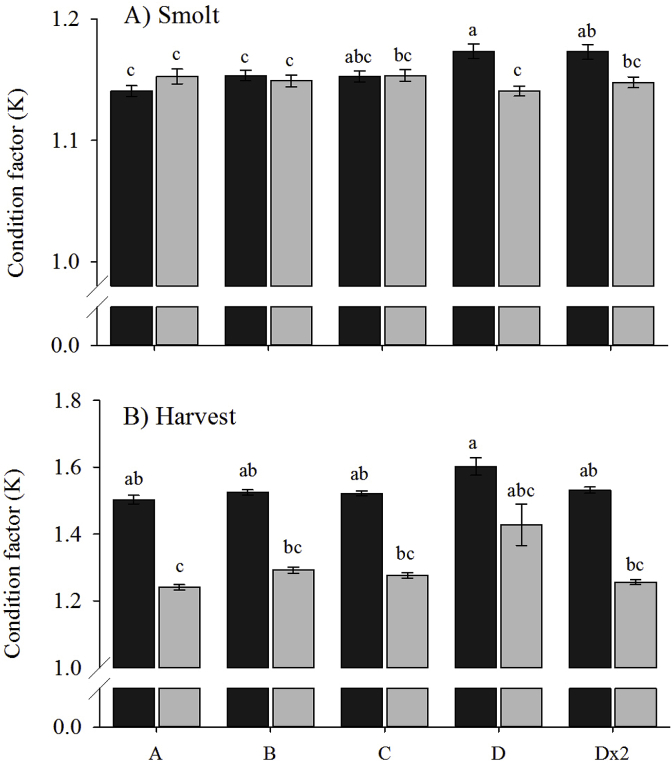
Condition factor (K) (mean ± SEM, n = 3) of diploid (black) and triploid (grey) Atlantic salmon in the five vaccine groups at A) smolt and B) harvest. Significant differences between ploidy and groups are indicated by different letters.

At smolt, no ploidy differences were observed for K, with the exception of Group D where diploids (1.17 ± 0.01) had significantly higher K than their triploid counterparts (1.14 ± 0.01) (Fig. 8A). Within the diploids, K in Groups D and Dx2 (1.17 ± 0.01) was significantly higher than Groups A (1.14 ± 0.01) and B (1.15 ± 0.01). No significant differences in K were found between vaccine groups in the triploids.

#### 3.4.2 Seawater phase

In May 2017 (6 months post-transfer to seawater), triploids (809.4 ± 14.4 g) were significantly heavier than their diploid counterparts (608.4 ± 12.6 g) in all five vaccine groups (Fig. 5C). Within the diploids, Groups A (645.2 ± 13.1 g), B (630.2 ± 11.3 g) and C (610.9 ± 12.1 g) were significantly heavier than Groups D (590.6 ± 12.1 g) and Dx2 (565.6 ± 147 g). Triploid fish in Group A (883.3 ± 14.8 g) were significantly heavier than those in Group Dx2 (729.7 ± 14.2 g). In August 2017, triploid weight (1962.6 ± 41.8 g) remained higher than that of the diploids (1646.4 ± 45.6 g), significantly so in Group A (triploids 2119.1 ± 39.9 g; diploids 1728.9 ± 48.9 g) (Fig. 5D). Within the diploid group, Group B (1728.8 ± 40.1 g) was significantly heavier than Group Dx2 (1505.1 ± 46.7 g). In the triploids, Groups A and B (2088.9 ± 46.2 g) were significantly heavier than Groups D (1857.7 ± 37.6 g) and Dx2 (1761.4 ± 48.5 g).

In October 2017 (11 months post-transfer to seawater), weight was not significantly affected by ploidy or vaccine group (Fig. 5E). At harvest (Jan 2018), diploids (4917.2 ± 137.3 g) weighed significantly more than their triploid counterparts (4579.4 ± 141.1 g) in all groups, except Group B (diploid 5013.2 ± 151.5 g; triploid 4886.2 ± 145.3 g) (Fig. 5F). Within both ploidy, vaccine group did not have a significant effect on weight.

Seawater TGC (smolt to harvest) was higher in diploids (2.98 ± 0.06) than in triploids (2.83 ± 0.03) for all vaccine groups, significantly so in Group C (diploids 3.02 ± 0.03; triploids 2.84 ± 0.02) (Fig. 6B). No correlation (*p* = 0.82) between TGC and adhesion score was found (Fig. 7B). Regression analysis showed that the vertical distance between the diploid and triploid slopes was not statistically significant (*p* = 0.07), with parallelism statistics (ANCOVA) revealing no significant difference in gradient (*p* > 0.05) between ploidy, which would indicate similar response to vaccination between ploidy.

At harvest, K in diploids (1.54 ± 0.01) was higher than their respective triploids (1.30 ± 0.02), significantly so in Group A (diploids 1.50 ± 0.01; triploids 1.24 ± 0.01) (Fig. 8B). No significant effect of vaccine groups was observed in both ploidy, vaccine group did not have a significant effect on K.

### 3.5 X-ray radiography for vertebral deformities

Vertebrae number did not differ significantly between diploids (57.8 ± 0.5) and triploids (57.7 ± 0.6) (*p* = 0.28). Prior to vaccination (“baseline”), the prevalence of moderate-severe deformities (>6 dV) was not significantly different between ploidy (diploids 2.0 ± 1.8%; triploids 6.0 ± 2.5%) (data not shown). Prevalence did not change from “baseline” to smolt (diploid 0.8 ± 0.5%; triploid 12.3 ± 4.5%) (*p* > 0.05), while it increased significantly from “baseline” to harvest in both diploids (15.9 ± 1.9%; *p* = 0.002) and triploids (34.9 ± 5.9%; *p* = 0.008).

**Fig. 9:**
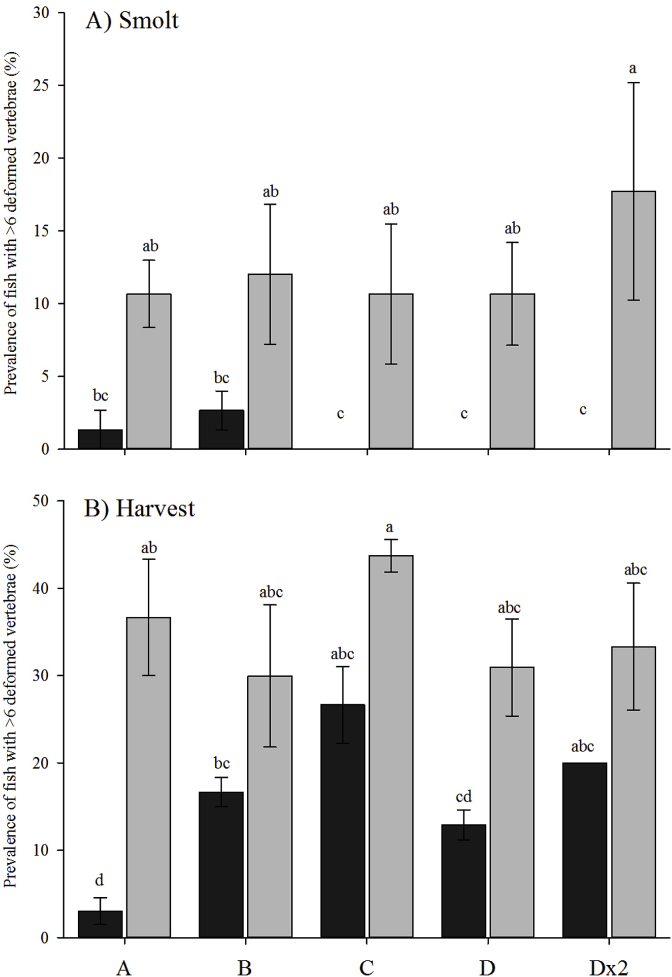
Prevalence (%) of individuals with 6 or more deformed vertebrae (dV) in diploid (black) and triploid (grey) Atlantic salmon at A) smolt and B) harvest (mean ± SEM, n = 3). Significant differences between ploidy and vaccine groups are indicated by different letters.

At smolt, ploidy significantly affected the prevalence of ≥6 dV (*p =* 0.00) with triploids consistently showing higher deformity prevalence than diploids, significantly so in Groups C, D and Dx2 (diploids 0%, triploids 10.7–17.2%) (Fig. 9A). Within both diploids and triploids at smolt, vaccine group did not significantly affect the prevalence of fish exhibiting ≥6 dV (*p =* 0.79).

Similarly, at harvest, triploids showed consistently higher deformity prevalence than diploids, although only significantly so in Group A (diploid 3.0 ± 1.5%; triploid 36.7 ± 6.7%) (Fig. 9B). In addition, within diploids, vaccine group had a significant effect on deformity prevalence with Group A exhibiting significantly lower deformity than Groups B (16.7 ± 1.7%), C (26.7 ± 4.4%) and Dx2 (20.0 ± 0.0%). No significant vaccine group effect was observed in the triploids.

Within the diploids, the prevalence of fish with ≥6 dV was significantly higher at harvest than at smolt in all vaccine groups (smolt 0.8 ± 0.5%, harvest 15.9 ± 1.9%), with the exception of Group A. Similarly, there was increased prevalence of fish with ≥6 dV from smolt to harvest in triploids. This was significant in Groups A (smolt 10.7 ± 2.7%; harvest 36.7 ± 6.7%) and C (smolt 10.7 ± 4.8%; harvest 43.7 ± 1.9%).

**Fig. 10:**
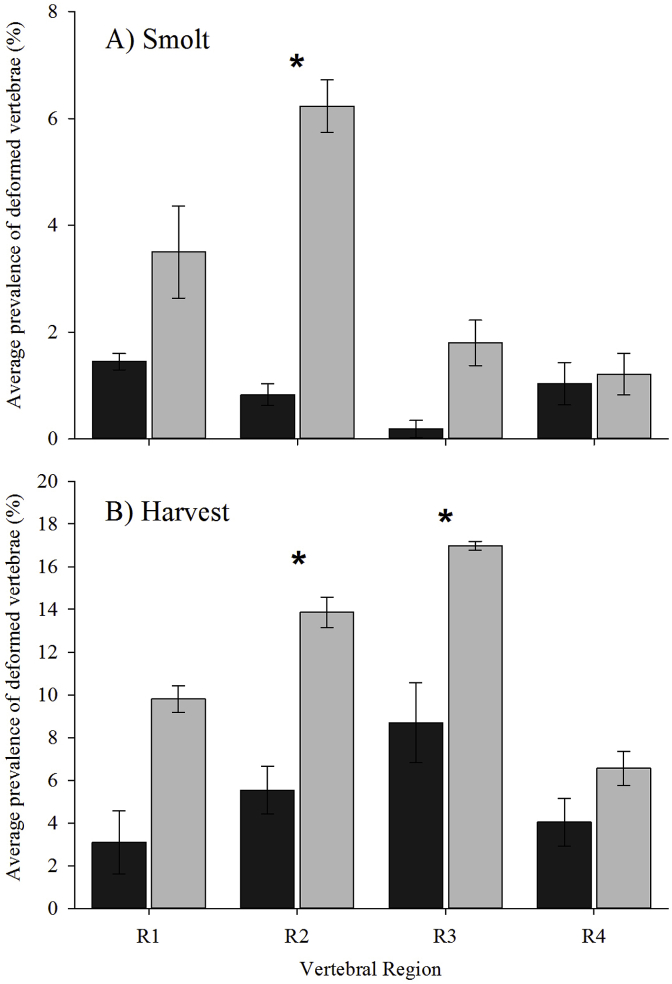
Average prevalence (all vaccine groups) of deformed vertebrae within each spinal region (%) in diploid (black) and triploid (grey) Atlantic salmon at A) baseline, B) smolt and C) harvest (mean ± SEM, n = 3). Asterisk (*) denotes significant differences between ploidy within vertebral regions.

Deformed vertebrae (dV) were observed in all four spinal regions in both ploidy at smolt and harvest (Fig. 10A and B). At smolt, triploids consistently had more deformed dV than diploids, significantly so in the caudal trunk (R2) (*p* = 0.010; triploids 6.23 ± 0.49%, diploids 0.83 ± 0.2%) (Fig. 10A). Triploids had the highest prevalence of dV in R2 while diploids showed the highest prevalence of dV in the cranial trunk (R1).

At harvest, triploids continued to exhibit more dV than diploids, again significantly in R2 (*p* = 0.008; triploids 13.86 ± 0.71%, diploids 5.53 ± 1.11%), as well as in the tail region (R3) (*p* = 0.048; triploids 16.97 ± 0.21%, diploids 8.69 ± 1.88%) (Fig. 10B). The region with the highest deformity prevalence in both ploidy was R3, with increased prevalence remaining in R2.

In terms of time changes, the prevalence of dV in R3 in diploids significantly increased from smolt to harvest (*p* = 0.046), with no significant time changes for the other regions. For triploids, there was a significant effect of time in all four regions, with higher prevalence observed at harvest than at smolt (*p* = 0.001–0.027).

**Fig. 11:**
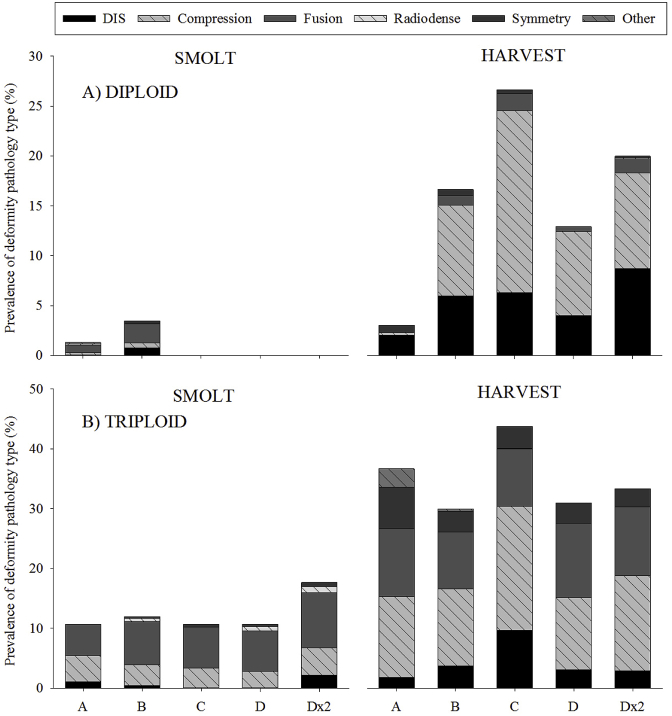
Prevalence (%) of different vertebral deformity pathology types (decreased intervertebral space, compression, fusion, radiodense, symmetry and other) within the deformed population with ≥6 dV of A) diploid and B) triploid Atlantic salmon at smolt and harvest.

In the diploids groups at smolt, while the prevalence of fish with ≥6 dV was low (0–2.7%), the most predominant vertebral deformity type in all groups was fusions (0.7–1.9%) followed by compressions (0.29–0.49%) (Fig. 11A). The most common type of fusion pathology was complete fusion (type 7) (0–1.5%) followed by fusion centre (type 8) (0–0.7%). In terms of compressions, the most prevalent type was compression and reduced intervertebral space (type 3) (0–0.4%) followed by one-sided compression (type 5) (0–0.3%). By harvest, the prevalence of all vertebral deformity types in all diploid vaccine groups had increased. A clear shift in deformity type prevalence was found at harvest in Groups B to Dx2, with compressions occurring most frequently (0.3–18.3%), followed by decreased intervertebral space (2.1–8.8%) and fusions (0–1.4%). The most common type of compression pathology was compression and decreased intervertebral space (0–15.4%), followed by one-sided compression (0.1–1.1%). In terms of fusion, the most prevalent type was complete fusion (0–0.9%), followed by compression and fusion (type 6) (0–0.9%).

As with the diploids at smolt, the most predominant vertebral deformity type in all triploid groups was fusions (5.3–9.2%), followed by compressions (2.8–4.6%) (Fig. 11B). The most common type of fusion pathology was fusion centre (3.0–4.9%) followed by complete fusion (0.7–2.8%). In terms of compressions, the most prevalent type was one-sided compression (2.7–4.4%), followed by compression without X structure (type 4) (0–0.4%). By harvest, the prevalence of all vertebral deformity types had increased from that exhibited at smolt. Compressions (12.1–20.7%) were found to be most prevalent deformity type in all of the triploid groups at harvest, followed by fusions (9.5–12.4%). The most common type of compression pathology was one-sided compression (9.9–11.9%), followed by compression and reduced intervertebral space (0.6–9.1%). In terms of fusion, the most prevalent type was fusion centre (3.9–8.2%), followed by complete fusion (0.7–4.6%).

## 4 Discussion

Vaccination, an essential tool for disease prevention, is now common practice in commercial salmonid aquaculture [53]. However, despite the desire to employ triploid Atlantic salmon in full-scale production to overcome pre-harvest maturation, published studies on triploid response to vaccination remain scarce. Considering this, the current study compared the response of diploid and triploid Atlantic salmon to commercially available vaccines, in terms of performance and the occurrence of side-effects.

Mortality during freshwater (vaccination to smolt) and seawater (smolt to harvest) generally remained below 10% in all vaccine groups, with no significant effect of ploidy. Comparable ploidy mortality concurs with previous research into triploid vaccination [42,43] as well as recent grow-out studies [33,45,[54], [55], [56], [57]]. It can be suggested that this is linked to advances in triploid nutrition [[31], [32], [33]] and environmental requirements [30,35,36,58,59]. However, higher mortality was observed in the triploid Dx2 group (16.8%) in seawater due to increased mortality in one of the triplicate cages (34.2% versus 7.7 and 8.6%). Suggestions to the cause of mortality cannot be drawn as no disease outbreaks were observed, and no nuisance seal activity was reported.

The measurement of antibody levels following the vaccination of Atlantic salmon is indicative of the immune response [60]. While the vaccines in this study contained a combination of antigens, *A. salmonicida* was used as it was the common antigen in all groups and has previously elicited a positive IgM response in salmonids [48,61,62]. Ploidy did not significantly affect antibody response to *A. salmonicida*, a finding consistent with previous studies in Atlantic salmon, ayu (*Plecoglossus altivelis*) and chinook salmon (*Oncorhynchus tshawytscha*) [38,63,64]. Vaccine group, however, did significantly affect antibody response. The vaccine-containing groups B, C, D and Dx2 showed a positive antibody response, which was significantly higher than the negative antibody response elicited by the PBS-control group (A). This is supported by previous research in which vaccination elicited an increased antibody response compared to sham-injections [38,61,64,65]. However, the antibody responses obtained in all the vaccine groups in the current study were lower than expected when compared to previous studies [60,61]. Using a whole cell *A. salmonicida* ELISA, 1/50 serum dilution and sampling at 7 weeks post-vaccination (conditions as per the current study), Erdal and Reitan [61] found that vaccinated fish exhibited an average antibody response of 1.16, three times greater than this study (diploid range 0.19–0.46; triploid range 0.22–0.42). The onset of protection for the vaccines utilised in this study is documented to occur between 520 and 600 °D post-vaccination so it could be suggested that, due to sampling occurring at 622 °D post-vaccination, the response was still in the early stages of development. Additionally, Erdal and Reitan [60] used constant 12 °C, while water temperature in this trial decreased from 16 °C at vaccination to 9 °C at sampling. As such, the decreasing temperature could have slowed down antibody development and a greater response may have been observed with higher and/or constant water temperature [66,67].

Intra-abdominal adhesions are the most common side-effect of the vaccination process and have the potential to cause severe muscle damage and carcass downgrading at harvest [18,22,43]. At smolt, results from ordinal logistic regression suggested that triploids were more likely to experience higher adhesion scores (“3” or “4”) than diploids. This finding concurs with a previous study assessing vaccination in S0+ smolts, which found significantly higher adhesion scores in triploids [43]. By harvest, triploids were not more likely to experience higher adhesion scores than diploids, a finding which is supported by studies in S1+ smolts [38] and harvest-size diploid and triploid Atlantic salmon [68] which found similar adhesion scores between ploidy. Taking these findings into account, further studies are recommended to determine the reason why triploid Atlantic salmon may be more prone to higher adhesion scores and to assess the interaction of temperature, vaccine type (antigens, adjuvants, manufacturer), vaccine dose and ploidy on the occurrence of adhesion.

At smolt and harvest, vaccine group significantly affected adhesion score in both diploids and triploids, with scores increasing as anticipated. This concurs with a previous study which showed increased adhesion scores with increasing antigen number [69]. Furthermore, while adhesion severity increased as expected, patterns remained similar over time. This is supported by Berg et al. [70] who found that adhesion scores in Atlantic salmon increased quickly following vaccination and then remained stable until harvest. The highest adhesion scores (“3” and “4”) were consistently found in the “accidental double-dose” group (Dx2) while the lowest (“0”) occurred in the PBS-control group (A). This demonstrates the negative effect that can be caused by improper vaccine administration and highlights the necessity for optimal vaccination strategies and practices [67].

The deposition of melanin in the viscera and musculature can result in down-grading at harvest [71]. Melanin deposits were observed in the viscera at smolt and harvest, with ordinal logistic regression suggesting that triploids were more likely to experience increased prevalence (%) of higher scores. This finding concurs with Larsen et al. [71], whose study showed increased prevalence of melanin deposits post-vaccination in triploid S0+ smolts. However, as Larsen et al. [71] did not score the severity of the melanin deposits, it is unclear how the prevlance of each score changed within their populations. As the current study and Larsen et al. [71] represent the only records of melanisation in triploids post-vaccination, further investigations are necessary to fully elucidate ploidy differences in the occurrence of melanin deposits post-vaccination. At smolt and harvest, melanin deposits were observed in the viscera of all vaccine groups in both ploidy. This concurs with previous findings which observed melanin deposits occurring in both vaccinated and sham-vaccinated Atlantic salmon [71,72]. However, it should be noted that higher melanin scores occurred in the vaccine-injected groups (B – Dx2) than in the PBS-control group (A), which, again, concurs with previous research [72].

The growth performance of triploids continues to be debated [45,55,73,74], with triploid growth following vaccination relatively uncharacterised. In this study, triploids in each vaccine group were consistently heavier than their respective diploids until October 2017, with diploids significantly heavier by harvest (January 2018). This concurs with results from other triploid vaccination studies in that, regardless of vaccination or smolt regime, triploids were heavier than diploids in freshwater and initial seawater stages (current study +19.3%; Fraser et al. [42], +10.6%; Fraser et al. [43], +8.2%) but diploids were heavier by harvest (current study +7.02%; Fraser et al. [43], +8.6%). In addition, the current findings also support performance studies where triploids were heavier in freshwater and early seawater stages but lost their weight advantage by harvest [33,35,39,45,56,75]. As previous studies in triploid Atlantic salmon have reported a negative link between high temperatures (≥12 °C) and feed intake and growth [35,36], it could be suggested that the high water temperature experienced in the current study from July to October 2017 (12.9–15.4 °C), negatively impacted triploid growth.

Vaccine group significantly affected weight in both ploidy, with weight consistently highest in Group A (PBS) and lowest in Group Dx2 (double-dose). This supports previous studies which found reduced growth in vaccinated fish compared to unvaccinated controls [18,42,43,69,70,76]. Combining increased adhesion scores and reduced growth in the Group Dx2 fish clearly demonstrates the negative effect of incorrect vaccine administration on production.

In terms of growth rate, TGC in triploids was lower than diploids at both smolt and harvest which is indicative of a slower growth rate in triploids. This finding agrees with previous studies [75,77,78] but contrasts with studies showing improved freshwater growth [28,45,55]. Knowing that triploids were heavier than diploids at vaccination suggesting faster growth rate from first feeding, reduced TGC in triploids may be the result of high temperatures (14–16 °C) experienced both pre- and post-vaccination. This is supported by Sambraus et al. [35] whose study found that increased temperatures (>15 °C) negatively affected feed intake in triploids and subsequently reduced growth. In seawater, lower triploid TGC is supported by previous studies where the early growth advantage experienced by triploids is lost [45,55,68,75,79]. With few studies showing that triploid growth can be sustained at a higher rate through to harvest [28,33], it is recommended that further work be undertaken to fully optimise the rearing of triploids in seawater to achieve maximum growth potential.

Freshwater TGC was negatively correlated with adhesion score severity, and thus vaccine group, in both ploidy which concurs with previous studies [68,69]. During the seawater phase (smolt – harvest), regression analysis showed that the negative impact of adhesion score on TGC was reduced. This could be linked to the earlier observation that adhesion scores did not increase significantly between smolt and harvest and, as a result, seawater growth was able to continue “normally”. This is an encouraging finding for salmon producers in that, although initial vaccination and adhesion development may impact growth, the overall effect diminishes with time thus allowing fish to perform normally.

Vertebral deformities have previously been reported in farmed and wild salmon with potential to cause welfare concerns and economic losses for the salmon industry [21]. For the purpose this study, only fish presenting 6 or more dV were considered for analysis, a decision which stems from work by Hansen et al. [44] suggesting that fish exhibiting less than 6 dV would not be significantly affected in terms of performance or welfare. Radiographic deformity (%, ≥6 dV) at smolt and harvest was significantly affected by ploidy, with triploids consistently exhibiting higher levels than diploids. This supports previous studies using S0+ Atlantic salmon, which found a higher deformity prevalence in triploids compared to their diploid siblings [[32], [33], [34]]. However, in terms of deformity at smolt, findings are in contrast to Fraser et al. [43] whose study found S0+ triploid smolts with equal or lower deformities than diploids. Considering the proposed relationship between growth rate and the prevalence of skeletal deformity [44,45], the authors hypothesised that elevated temperatures in S0+ smolt production (16 °C for 42 days) impeded growth rates in triploids and as such, alleviated the risk of deformity. When TGC between vaccination and smolt transfer is calculated for S0+ smolts in the Fraser et al. [43] study, this hypothesis can be supported as triploid TGC (average 0.83) was lower than expected for this life-stage (~1.40, Liu et al. [80]). While an extended period of high temperature in the current study may have resulted in the same pattern, temperature decreased post-vaccination and freshwater TGC in triploids (0.88–1.12) was higher than that of Fraser et al. [43] and closer to expected [80]. So, despite lower TGC than diploids, triploids in the current study were still growing fast which may explain their significantly increased prevalence of vertebral deformities. To fully confirm this, it is recommended that research be undertaken to assess the effect of a range of high water temperatures for varying durations, in combination with smolt regimes and vaccination, on the development of vertebral deformities in triploid Atlantic salmon.

At smolt and harvest, vaccine group did not significantly affect radiological deformity in diploids and triploids, a finding which concurs with Fraser et al. [43] who found similar levels of deformity in unvaccinated and vaccinated Atlantic salmon. Moreover, the finding would appear to refute the suggestion that the development of vertebral deformities can be aggravated through the vaccination process [21]. In both ploidy, deformity prevalence increased over time from smolt to harvest which concurs with previous studies [32,33]. Elevated seawater temperatures prior to harvest (July–October 2017; 12.9–15.4 °C) may have aggravated deformity prevalence but further studies would be required to elucidate the effect of a seawater “temperature spike” on the prevalence of vertebral deformities.

Deformed vertebrae were found in the four vertebral regions in both ploidy at smolt and harvest. Within R2 at smolt, and R2 and R3 at harvest, triploids had significantly higher prevalence of dV than diploids which supports previous reports [32,33]. In both ploidy at smolt, R2 had the highest deformity prevalence which concurs with previous studies indicating this as the most affected vertebral region during freshwater growth [32,54,81,82]. By harvest, the region exhibiting the highest deformity prevalence in both ploidy had shifted from R2 to R3, again supporting previous research [27,32,33,43,83]. The types of dV pathology within the deformed population were also assessed. At smolt and harvest, fusions (type 6–8) and compressions (type 2–5) were the most prevalent pathology types in all ploidy vaccine groups in line with previously published studies [30,32,43,45,81]. Collectively, deformity results from the current study suggest that ploidy, in conjunction with other factors (e.g. temperature profile) rather than vaccine, is predisposing triploids to increased risk of vertebral deformities.

## 5 Conclusions

This study showed that triploids respond as well as diploids to vaccination. Similar antibody responses indicate that all the vaccines assessed would be as effective in protecting triploids as diploids following disease challenge. Ploidy significantly affected both adhesion and melanin scores, suggesting that triploids may be prone to higher scores than diploids, although further studies would be required to fully elucidate these differences. The significant weight difference maintained by triploids throughout freshwater and early seawater was lost at harvest which suggests that further studies are required to establish the full growth potential of triploids and determine optimum farming locations for the deployment of triploid Atlantic salmon. The significant effect of vaccine group and adhesion score on TGC highlights the need for accurate commercial vaccination procedures. It should be noted that Group Dx2 is not commercial practice and was used only to demonstrate the negative effect of “accidental double-dosing” on fish performance and welfare. As in previous studies, vertebral deformities remained higher in triploids than in diploids. This indicates the need for further studies to advance the current knowledge of triploid nutrition and thermal requirements and thus fully elucidate the issue of triploid vertebral deformities. Overall, the findings support the use of commercial vaccines in triploid Atlantic salmon, and continue to support the suitability of triploids for commercial-scale salmon production.
